# Effects of Caffeine Ingestion on Human Standing Balance: A Systematic Review of Placebo-Controlled Trials

**DOI:** 10.3390/nu13103527

**Published:** 2021-10-08

**Authors:** Isobel Briggs, Joel B. Chidley, Corinna Chidley, Callum J. Osler

**Affiliations:** Human Sciences Research Centre, University of Derby, Kedleston Road, Derby DE22 1GB, UK; i.briggs1@unimail.derby.ac.uk (I.B.); J.Chidley@derby.ac.uk (J.B.C.); C.Chidley@derby.ac.uk (C.C.)

**Keywords:** human, caffeine, balance, postural control, ageing

## Abstract

Caffeine ingestion may influence balance control via numerous mechanisms. Although previously investigated using various study designs and methods, here we aimed to create the first evidence-based consensus regarding the effects of caffeine on the control of upright stance via systematic review (PROSPERO registration CRD42021226939). Embase, PubMed/MEDLINE, SPORTDiscus and Web of Science databases were searched on 27 January 2021 to identify placebo-controlled trials investigating caffeine-induced changes in human standing balance. Reference lists of eligible studies were also searched. Overall, nine studies involving a total of 290 participants were included. All studies were moderate to strong in quality according to the QualSyst tool. Balance-related outcome measures were collected across a range of different participant ages, stances and sensory conditions. The results show that younger participants’ balance was generally unaffected by caffeine ingestion. However, a significant balance impairment was observed following caffeine ingestion in all studies involving older participants (average age >65 years). Our results therefore suggest an age-dependent effect of caffeine ingestion on human standing. Further research into this effect is warranted as only one study has directly compared younger and older adults. Nonetheless, an important implication of our findings is that caffeine ingestion may increase fall risk in older adults. Furthermore, based on our findings, caffeine ingestion should be considered as a potential confounding factor when assessing human standing balance, particularly in older adults.

## 1. Introduction

Caffeine (1,3,7-trimethylxanthine) is the most commonly consumed stimulant worldwide [1]. It is hydrophilic, distributing freely into intracellular tissue water [2] whilst also being sufficiently lipophilic to pass through all biological membranes and readily crosses the blood–brain barrier [3,4]. Following ingestion, it is rapidly absorbed by the body and appears in the blood within 5–15 min, with serum concentration peaking between 40 and 80 min [5]. Caffeine mediates many of its physiological actions through the antagonism of central adenosine receptors [6]. As adenosine is an inhibitory neuromodulator in the central nervous system with sedative-like properties, caffeine blocking of adenosine has several desirable effects at low to moderate doses, including changes in mood, energy, alertness and vigour [6,7]. Furthermore, at a peripheral level, there is evidence to suggest that caffeine can directly enhance skeletal muscle function [8]. The net effect of these central and peripheral mechanisms of caffeine is the potential for improvements in a wide range of cognitive and physical functions [9].

One sensorimotor function potentially influenced by caffeine ingestion is the control of human standing. Maintaining an upright stance requires appropriate activation of postural muscles in response to integrated sensory feedback or in anticipation of a balance disturbance. Although seemingly automatic and, to an extent, controlled via unconscious balance mechanisms, evidence suggests that cortical structures and cognitive processes are also involved [10,11]. Therefore, since the control of balance is dependent on lower limb muscle function and amenable to cognitive influence, it is interesting to speculate whether caffeine ingestion impacts balance performance. On the one hand, enhanced muscular function [12] and aspects of cognition—including attention and perception [13] —following caffeine ingestion could improve balance control. On the other hand, caffeine ingestion could impair balance via an alternative mechanism, such as postural disturbances associated with its stimulating effects on ventilation [14]. Therefore, while this effect is poorly understood at present, it is clear that caffeine has the potential to modulate the control of human balance via numerous physical and cognitive mechanisms.

The control of human balance is particularly important to the world’s ageing population, with one in three older adults experiencing a fall each year [15]. Many factors are widely accepted to contribute to poor balance and falls in older adults, such as sensory loss, muscle weakness and cognitive decline [16]. Age-related changes to the cortical control of balance have also been reported [17]. However, the role of nutrition has received considerably less attention. Interestingly, ageing has been shown to influence several of the aforementioned candidate mechanisms linking caffeine ingestion to balance control. For example, past research has found age to modulate the performance enhancing effect of caffeine on muscle [18] and cognitive [19] function. Furthermore, a reduced rate of caffeine metabolism with increasing age has been postulated [20]. This evidence raises the possibility that the effect of caffeine ingestion on balance (if any) may be different in older adults, which could be of importance to the aetiology of falls in this population.

The effects of caffeine ingestion on balance control have been investigated since the middle of the last century [21]. These early researchers conducted a series of experiments in fatigued and/or sleep-deprived military personnel. However, participants were reported to have ingested a combined 7.5 g capsule of caffeine and sodium benzoate, making the precise dose of caffeine unclear. Research has continued right up to the present day using a variety of methods, study designs and populations, including older adults. However, there is currently no systematic review available. We therefore aimed to create the first evidence-based consensus regarding this effect by synthesising results of only placebo-controlled studies. Furthermore, given the potential for changes in both the control of balance and responsiveness to caffeine in older adults, we also aimed to investigate a possible age-dependent effect of caffeine ingestion on human balance control.

## 2. Materials and Methods

The Preferred Reporting Items for Systematic Reviews and Meta-Analyses (PRISMA) guidance was followed when conducting this systematic review [22,23].

### 2.1. Eligibility Criteria 

Eligibility criteria were formulated using the PICOS (i.e., Population, Intervention, Comparison, Outcome, and Study design) method (Table 1). Only studies involving human participants were included. This included studies conducted in adults across the entire lifespan, although findings for older adults were synthesised separately. Studies involving patient groups were not excluded. To be included, studies had to compare caffeine ingestion in any form (e.g., drink, capsule) with ingestion of a placebo control. Studies that failed to use a taste and/or sight matched control condition were excluded. Only studies measuring balance control during upright stance (e.g., posturography) were included. Dynamic standing tasks (e.g., on a moving platform) were permitted, but balance tests involving a concurrent voluntary movement (e.g., step initiation) were excluded. The necessity for a placebo condition/group meant that only placebo-controlled studies were included, although the study design could be crossover or parallel. Furthermore, studies were included whether randomised, non-randomised or not specified. Studies that made only a before–after caffeine comparison were excluded. All had to be original research studies published in a peer-reviewed journal. There were no restrictions on language or year of publication.

### 2.2. Information Sources, Search Strategy and Selection Process

On 27 January 2021, Embase, PubMed/MEDLINE, SPORTDiscus and Web of Science databases were searched using terms relating to caffeine and balance control. The search strategy development process is shown in Table 2, with the final search terms being: (“Caffeine” OR “Caffeinated” OR “Energy drinks” OR “Energy drink” OR “Coffee”) AND (“Balance” OR “Postural stability” OR “Postural sway” OR “Static balance” OR “Posture” OR “Postural control” OR “Standing” OR “Upright stance”). For Embase, PubMed/MEDLINE and Web of Science, all database fields were searched (i.e., All Fields option). This option was not available in SPORTDiscus, so the default field search was used. Embase and Web of Science searches were limited to only Embase and Web of Science Core Collection databases, respectively. In accordance with the eligibility criteria, searches were not restricted based on language nor were searches restricted based on year of publication, meaning the full dates of coverage for each database were searched.

After removing duplicate search results between databases, potentially eligible studies were identified by screening titles and abstracts. Full copies of these studies were then obtained to assess against the aforementioned eligibility criteria. Screening and full text review were completed independently by the two lead review authors (I.B. and C.J.O.), with disagreements resolved by discussion and, if necessary, referral to the remaining authors (J.B.C. and C.C.). I.B. and C.J.O. then independently examined the reference lists of all included studies to identify additional eligible studies.

### 2.3. Study Quality and Risk of Bias Assessment

The quality and risk of bias of individual studies were assessed using the QualSyst tool [24]. This consisted of fourteen items (see Table 3) each rated on the degree to which criteria were met (0 = no, 1 = partial, 2 = yes). A percentage score was then calculated which was used to determine the quality of studies and was considered when interpreting results, rather than used as an inclusion/exclusion criterion. Scores of ≥55% and ≥75% were used as thresholds to indicate moderate and strong quality, respectively. I.B. and C.J.O. independently conducted this assessment and, as for study selection, disagreements were resolved by discussion. 

### 2.4. Data Collection and Synthesis

The following data items were sought from included studies: authors, year of publication, corresponding author affiliation, sample size and characteristics, study design, pre-experiment caffeine abstinence protocol, dose and timing of caffeine/placebo, and balance-related outcome measures. In accordance with the eligibility criteria, any measure of balance control during upright standing was included. It is common for studies to measure balance under various conditions, such as different stances (e.g., bipedal/semi-tandem) and/or sensory conditions (e.g., eyes open/closed; firm/foam support surface). Furthermore, we anticipated that some studies would assess balance at various time points during the acute period following caffeine ingestion (e.g., 60, 120 and 180 min). We therefore placed no restriction on the number of balance measures and/or time points; data were collected for all eligible measures. This included cases where studies reported an overall test score providing a composite measure of balance function. If stance and support surface were not reported, we then assumed bipedal stance and firm support surface, respectively.

I.B. extracted all relevant data from the included studies. This was then checked by C.J.O., with any discrepancies being resolved through discussion. Where necessary, I.B. contacted corresponding authors of the included studies to clarify study details and/or request unreported data. Where data could not be obtained in this way, numerical data were extracted from published figures using the WebPlotDigitizer software (Version 4.4, Automeris LLC, Pacifica, CA, USA). Group average data were used to calculate the percentage difference between caffeine and placebo conditions for data synthesis and tabulation. In accordance with our aim, a separate data synthesis and summary table were used for participant groups with an average age of 65 years or over to illustrate potential age-dependent effects. Included studies are ordered by year of publication within tables. A meta-analysis was not undertaken due to the heterogeneity of design and method within the included studies.

## 3. Results 

### 3.1. Study Selection

**Figure 1:**
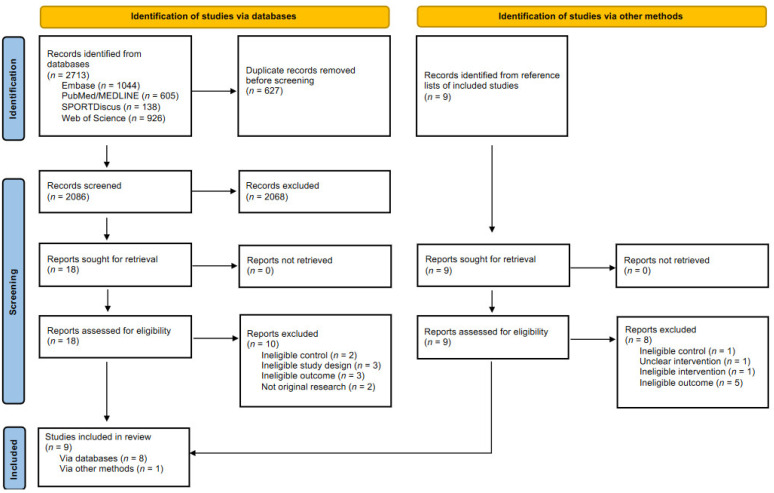
PRISMA 2020 flow diagram for new systematic reviews which included searches of databases and other methods [22].

As shown in Figure 1, initial database searches resulted in a total of 2713 records, of which 2086 remained after duplicates were removed. Eighteen records remained after screening for eligibility by means of study title and abstract. Following assessment of these eighteen full articles against the eligibility criteria, a further ten were excluded. Reference list screening resulted in one additional study, meaning a total of nine eligible studies were included in the systematic review [25,26,27,28,29,30,31,32,33].

Some studies which investigated the effects of caffeine on human balance were not included because the full inclusion criteria were not met. Of note, three studies were excluded for using a non-placebo control (i.e., coffee vs. caffeine abstinence [34]; Red Bull vs. Squirt citrus-flavoured soft drink [35]; caffeine powder mixed with water vs. water only [36]). A further three were excluded for using a before–after caffeine study design with no control condition [37,38,39]. One was excluded for an unclear caffeine intervention (i.e., 7.5 g caffeine sodium benzoate capsule [21]) and one was excluded for investigating the effects on balance of caffeine only when combined with alcohol [40].

### 3.2. Study Quality, Risk of Bias and Characteristics

Of the nine eligible studies, five investigated the effects of caffeine on human standing balance in a participant group aged less than 65 years (i.e., younger) [25,26,27,28,32]. Five studies included a participant group with an average age of 65 years or over (i.e., older) [27,29,30,31,33]. Swift and Tiplady (1988) was the only study to compare groups of younger and older participants [27].

#### 3.2.1. Younger

Three of five studies including younger participant groups were of moderate quality [25,26,27] and the remaining two were of strong quality [28,32] (see Table 3). Franks et al. (1975) was the only study that was not stated to be double-blind; although participants were described as “ignorant” to their treatment group, this study did not mention blinding of the experimenter [25]. Of note, while four of the five studies stated that participants were randomly allocated to group/condition, Ben Waer et al. (2020) did not report randomisation or counterbalancing [32]. Swift and Tiplady (1988) included the smallest sample size of all studies, with six participants in each age group, and also failed to report variance for some non-significant results [27].

Table 4 summarises the studies conducted in younger participants. These five studies were affiliated to five separate laboratories, each in a different country. A total of 124 healthy adults participated, including 49 males and 75 females. Four of the five studies used a crossover study design, with Franks et al. (1975) the only parallel study [25]. The studies which reported the duration of caffeine abstinence prior to testing used a period of between 12 and 24 h [26,28,32], but Franks et al. (1975) did not report the duration which participants abstained from caffeinated beverages [25] and Swift and Tiplady (1988) made no mention of pre-experiment caffeine abstinence [27]. Four of five studies used an absolute caffeine dose of between 100 and 500 mg [26,27,28,32], with the 4.3 mg∙kg^−1^ (i.e., relative) dose used by Franks et al. (1975) also likely to fall within this range [25]. Three studies used caffeine in capsule form [27,28,32] and two studies added caffeine to decaffeinated coffee [25,26]. In all cases, the form of the caffeine and placebo conditions were matched. Timing of the balance measurement following caffeine ingestion also varied from 20 to 180 min. Three studies elected to assess balance at multiple time points across a period of 2–3 h post-ingestion [25,26,27], whereas two studies used a single balance measurement at 30–45 min[28,32].

#### 3.2.2. Older

Four of five studies including an older participant group were of strong quality [29,30,31,33]. As outlined above, Swift and Tiplady (1988) was rated as moderate quality [27] (see Table 3). 

Table 5 summarises the studies conducted in older participants. Two studies were affiliated to separate UK laboratories [27,33], while the remaining three were all conducted by the same group of researchers in Denmark (i.e., Regional Hospital Herning/Aarhus University Hospital) [29,30,31]. Four of five studies included healthy participants; the only exception was the study by Momsen et al. (2010), which studied intermittent claudication patients [30]. In total, 166 older adults participated, including 87 males and 79 females. All five studies were randomised double-blind crossover trials. As mentioned above, Swift and Tiplady (1988) did not state a time period for participants to abstain from caffeine prior to the experiment [27]. The remaining studies used a pre-experiment abstinence period of between 8 and 48 h [29,30,31,33]. Swift and Tiplady (1988) used a caffeine dose of 200 mg [27], which was, on average, equivalent to the 3 mg∙kg^−1^ dose used by Tallis et al. (2020) [33]. The remaining three studies used a relatively high caffeine dose of 6 mg∙kg^−1^. All studies used capsule ingestion for both caffeine and placebo conditions. While Swift and Tiplady (1988) tested balance at 60, 120 and 180 min post-ingestion [27], the remaining four studies tested balance once at 45–75 min [29,30,31,33].

### 3.3. Study Findings

#### 3.3.1. Younger

All five studies in younger adults included the assessment of balance when standing with eyes open on a firm surface (EO). Four of these studies found caffeine to have no effect on balance under these conditions when assessed 30–180 min following a dose of between 100 and 500 mg [26,27,28,32]. This included the study by Liguori and Robinson (2001), where an EO condition was part of the EquiTest protocol used, though a composite score based on all six conditions was reported rather than findings for each condition [28]. Results of the remaining study, by Franks et al. (1975), showed a 27% increase in body sway compared to placebo when measured 20 min following ingestion of 4.3 mg∙kg^−1^ of caffeine. However, even in this study, there was no significant effect at 80 and 140 min post-ingestion [25]. Four of these studies also investigated the effects of caffeine ingestion on balance when standing on a firm surface with eyes closed (EC); all showed no significant effect under these conditions [25,26,28,32]. Liguori and Robinson (2001) also included a further condition standing on a firm surface with a visual surround that moved with the body (i.e., sway-referenced); again, no significant effect of caffeine ingestion was reported, although based on only a composite score of six conditions in total [28].

Two studies assessed the effects of caffeine on balance during conditions designed to reduce proprioceptive information regarding body sway [28,32]. The EquiTest protocol used by Liguori and Robinson (2001) included conditions with a sway-referenced support surface (completed with eyes open, eyes closed, and a sway-referenced visual surround). The reported composite score data showed no significant effect 45 min following a caffeine dose of 200 or 400 mg [28]. Ben Waer et al. (2020) used a 134 mm thick foam surface (completed with eyes open (EOF) and closed (ECF)). In the EOF condition, they found no significant effect on standing balance 30 min following a caffeine dose of 100 or 400 mg. In the ECF condition, several measures of body sway derived from centre of pressure recordings were significantly reduced by 16% following 100 mg of caffeine compared to placebo, but there was no significant effect in the 400 mg condition [32].

#### 3.3.2. Older

All five studies within older participants included an EO condition, and all five found at least one post-caffeine measure of body sway to be increased relative to placebo under these conditions [27,29,30,31,33]. Swift and Tiplady (1988) showed a significant increase in anteroposterior body sway in older adults at 180 min post-ingestion of 200 mg caffeine, but there was no significant effect at 60 or 120 min [27]. Three studies using a 6 mg∙kg^−1^ dose found a centre of pressure-derived measure of body sway to be increased by 19–25% when measured 60–75 min post-ingestion [29,30,31]. Tallis et al. (2020) reported five different centre of pressure-derived measures taken 45 min following 3 mg∙kg^−1^ of caffeine; path length and mean velocity increased by 21–22% and maximal mediolateral displacement increased by 89%, while anteroposterior and elliptical area measures were not significantly affected [33]. Four of the five studies also included an EC condition [29,30,31,33]. The three studies using a 6 mg∙kg^−1^ dose found a centre of pressure-derived measure of body sway to be increased by 22–43% [29,30,31]. Tallis et al. (2020) once again found no significant effect of caffeine ingestion on anteroposterior and elliptical area measures, but centre of pressure path length and mean velocity increased by 25–27% and maximal mediolateral displacement increased by 114% [33]. On a firm surface, two studies also investigated caffeine-induced changes in balance when standing in a semi-tandem stance (EOST), with both showing no significant effect 60 min following a 6 mg∙kg^−1^ dose [29,31].

One study in older adults also used a compliant foam standing surface (i.e., EOF and ECF conditions), reporting a similar pattern of results to the firm surface conditions [33]. That is to say, caffeine ingestion significantly increased the same three of five measures of body sway; centre of pressure path length and mean velocity increased by 6–8% and maximal mediolateral displacement increased by 23–34%.

Tallis et al. (2020) was also the only study to investigate the effects of caffeine on balance when participants performed a concurrent cognitive task (i.e., a dual task) [33]. For the duration of the standing trial, participants were required to count backwards aloud in threes (serial threes subtraction task) or sevens (serial sevens subtraction task), starting from a random three-digit number. These dual-task trials were completed on both firm and foam surfaces, always with eyes open. Statistically, the effect of caffeine ingestion was not different to other types of trial described above, with significant increases in the same three measures of body sway; centre of pressure path length and mean velocity changed by 2–8% and −6–9% on firm and foam surfaces, respectively. Maximal mediolateral displacement increased by 9–93% and 2–6% on firm and foam surfaces, respectively.

## 4. Discussion

The current study is the first systematic review to investigate the effects of caffeine ingestion on human standing balance. Our findings indicate that existing placebo-controlled trials have found caffeine to generally induce no change in the control of upright stance in younger adults. However, all of the studies which included older participants found balance to be significantly impaired following caffeine ingestion, with increases in body sway of up to 114% within this age group. There was only one instance where caffeine significantly improved balance, under the specific conditions of standing with eyes closed on a foam surface following a relatively low caffeine dose.

### 4.1. Age-Dependent Effect of Caffeine Ingestion on Human Standing Balance

The vast majority of results found caffeine ingestion to not affect younger adults’ balance when standing on a firm surface. Only the study by Franks et al. (1975)—which was rated as moderate in quality—reported a significant increase in sway 20 min following caffeine ingestion but not at 80 and 140 min [25]. While caffeine appears in the blood within 5–15 min and may have influenced balance in this time frame, it is difficult to explain why their findings showed no significant effect at 80 min post-ingestion considering that the peak plasma caffeine concentration occurs between 40 and 80 min [5]. No other study measured balance as soon as 20 min following ingestion, making it difficult to compare findings between studies. However, the caffeine abstinence protocol adopted in this study may be of importance in explaining the findings; while participants were asked to abstain from caffeinated beverages before arrival to the laboratory, a time frame was not reported and there was no mention of caffeine-containing foods. If caffeine was consumed in the hours before arrival and summed with the 4.3 mg∙kg^−1^ dose given as part of the experiment, this could have potentially led to a relatively high dose of caffeine in this particular study, which could explain the difference in findings. Whatever the reason for this individual result, it remains the case that all other studies in younger adults—many rated as strong in quality—were in agreement that caffeine induced no change in balance when standing on a firm surface [26,27,28,32]. This is also supported by numerous studies which did not meet the full criteria for this review [34,35,38], meaning we are confident that caffeine ingestion has little to no effect on standing balance in younger adults under these conditions.

In contrast to younger adults, older adults’ balance when standing on a firm surface was significantly impaired following caffeine ingestion in all studies included within the current systematic review [27,29,30,31,33]. Measures incorporating total body sway in the horizontal plane (i.e., mediolateral and anteroposterior) and measures of purely mediolateral sway were most affected [29,30,31,33], whereas anteroposterior measures were less frequently affected [27,33]. Furthermore, balance when standing in a semi-tandem stance was not significantly affected following caffeine ingestion [29,31]. To date, only the study by Swift and Tiplady (1988) has directly compared the effects of caffeine on balance control in younger and older participants [27]. While their findings demonstrated a significant caffeine-related balance impairment at 180 min post-ingestion in only older adults, numerous factors limit the confidence we have in this finding. Firstly, the study was rated as only moderate in quality. Secondly, only anteroposterior body sway was measured. Thirdly, only six participants were in each age group. Finally, significant impairment was not found at 60 and 120 min. Nonetheless, despite the lack of strong research directly comparing age groups, the overall findings of this systematic review do strongly suggest an age-dependent effect of caffeine ingestion on human standing balance, whereby older adults’ balance is more negatively affected compared to younger adults.

The mechanism which underlies the caffeine-induced balance impairment in older adults is currently unclear. Enhanced muscular and cognitive function [9,12,13] would be assumed to improve balance performance, the reverse of what we found in older adults. Nonetheless, greater cortical contribution to standing has been suggested in older adults [41], meaning the balance control system would be more amenable to a cognitive mechanism in this population. The role of cognition was investigated by only Tallis et al. (2020) [33]. These researchers asked older participants to concurrently perform a backwards counting task during the balance assessment. Close inspection of their data suggests that the addition of the cognitive task may attenuate the effects of caffeine on balance, with the average caffeine-induced balance impairment reduced (or even reversed) in many cases. This effect was not statistically significant perhaps on account of the relatively small sample size and associated type II error. Nonetheless, their findings do raise the possibility of an interaction between caffeine and cognition in the context of human balance control. In terms of mechanisms that may underlie the impairment seen in older adults, there are numerous candidates: increased ventilation, jitters, irritability, restlessness, and dizziness have all been suggested to occur following caffeine ingestion and could, in theory, bring about increased body sway [14,29,33]. A study by Polasek et al. (2013) reported a 33% reduction in the rate of caffeine metabolism in older (65–85 years) compared to younger adults (20–40 years), which may prolong some of the undesirable side effects and negatively impact balance in older populations in studies whereby measurements have been undertaken more than ~40–80 min following consumption [20]. However, past research has not been designed to investigate these theories, making discussion of underlying mechanisms here very speculative.

### 4.2. Possible Caffeine-Induced Balance Enhancement in Specific Conditions

While standing on a firm surface was shown to be unaffected and impaired by caffeine ingestion in younger and older adults, respectively, one study did demonstrate improved balance control under certain conditions [32]. This study, which investigated healthy middle-aged women, found enhanced balance control specifically when standing with eyes closed on a foam surface following 100 mg of caffeine. Although this finding alone provides insufficient evidence on which to base strong conclusions—particularly as the same study found no significant effect following 400 mg of caffeine—other research which did not meet the full criteria for our systematic review also points towards improved balance control under similarly challenging sensory conditions; two studies found caffeine to enhance balance when standing with eyes closed on a sway-referenced surface [34,37] and a further study found balance to be improved when standing on one leg on a moving platform [36].

In more challenging conditions, such as standing on a foam or moving surface, the aforementioned effects of caffeine ingestion on muscular and cognitive performance may be important due to increased involvement of these functions [42,43]. Alternatively, as the effects appear to depend on the sensory conditions, caffeine-induced changes to the processing and/or integration of balance-related sensory input is a plausible mechanism. Upright stance is maintained via postural adjustments generated when a disturbance to body position is either signalled by integrated sensory feedback (i.e., vestibular, visual and proprioceptive) or anticipated. The postural adjustment, in turn, influences the sensory feedback of body position, meaning the balance control system is a “closed loop” [44]. Therefore, to investigate changes to the processing of each underlying sensory input, balance can be disturbed at different places in the closed-loop system (e.g., electrical vestibular stimulation, visual scene movement, ankle rotations). While some studies included in this review removed or reduced sensory input (e.g., eyes closed, foam surface), no study disturbed balance via this type of perturbation. As balance improvements were found only when reduced availability of visual and proprioceptive feedback would have increased vestibular weighting [45,46], it is possible that a vestibular mechanism could underlie this effect. Although past research has shown caffeine ingestion to have minimal effects on commonly used tests of vestibular function [47,48,49], the effect on the vestibular-evoked balance response has not been investigated. It would therefore be of interest to use electrical vestibular stimulation to test the effects of caffeine ingestion on the vestibular control of balance [50,51], as the mechanism underlying enhanced balance in specific sensory conditions is currently unclear.

### 4.3. Limitations and Future Research

Although this systematic review provides insight into the effects of caffeine ingestion on human standing balance, the included studies are not without limitations. Interestingly, the three earliest papers (i.e., those published between 1975 and 1988; [25,26,27]) were rated as moderate as part of the quality assessment, whereas the six more recent papers were rated as strong [28,29,30,31,32,33]. This may be due to changes in terms of methodological rigour or scientific reporting standards over recent decades. Sample sizes were small in some studies, and future research should ensure an appropriate level of statistical power. Furthermore, aspects of the research design in some studies may have introduced bias; we recommend that randomised double-blind placebo-controlled trials are used in future research investigating the impact of caffeine ingestion on balance.

As mentioned above, caffeine-induced effects on balance have been directly compared between younger and older participants only once, and this study had numerous limitations including an inadequate sample size [27]. Therefore, we recommend that a larger trial is conducted to experimentally confirm the age-dependent effect of caffeine on balance suggested by our systematic review findings. Research to date has also failed to investigate how age modulates this effect over the entire lifespan. Concerning this point, a middle-aged group of participants was included in only the study by Ben Waer et al. (2020), but the lack of younger or older groups meant comparison across ages was not possible [32]. Furthermore, only one included study examined a patient group [30], with all others recruiting healthy participants. Investigating the effects of caffeine ingestion on balance control in less healthy individuals and/or those at higher fall risk would, therefore, be a logical direction for future research. 

Heterogeneity of methodology made comparison across the included studies more difficult to interpret. The nine studies utilised a range of caffeine abstinence protocols and doses. Norgaer et al. (2005) [29] and Jensen et al. (2011) [31] used 48- and 8-h abstinence periods, respectively, finding very similar levels of balance impairment. Based on the notion that 8 h would not be long enough to elicit withdrawal symptoms [31], caffeine ingestion, therefore, appears to influence balance control directly, although comparisons between different abstinence periods have not yet been made within the same study. In terms of the caffeine ingestion, only two studies compared more than one dose, and in these studies [28,32] as well as others [26,27], doses were administered as an absolute, as opposed to relative (i.e., mg∙kg^−1^), dose which may not account for differences in body mass between participants. One study found the effects to be no different between 200 and 400 mg of caffeine [28], whereas another study found enhanced balance following 100 mg but not following 400 mg of caffeine [32]. This raises the possibility that low doses have a more positive effect on balance control, but further research is needed to investigate the dose–response relationship. Our finding of impaired balance in older adults is based on studies using a caffeine dose of ~200 mg or greater. Therefore, future research should investigate the impact on balance control of lower caffeine doses. Another important consideration is that only three studies provided details of a manipulation check to test the validity of the placebo [29,30,31], but this is advised for future research.

Various measures of balance were also used. Balance is often assessed in different stances and sensory conditions. Furthermore, there are numerous different variables available to quantify an individual’s body sway. This, once again, made comparison across studies difficult to interpret, and it is important to consider that these variables are not always equivalent. We advise researchers to be clear on exactly how variables of body sway are calculated to aid interpretation of data, particularly when comparing across studies. Although the included studies used various traditional linear measures of body sway, none used nonlinear analysis methods. It would therefore be interesting to also use measures such as sample entropy to characterize the regularity of sway in future research [52]. Furthermore, in addition to measures of body sway during unperturbed stance, we also suggest that the balance response to sensory and physical perturbations is used in future research to investigate the mechanism(s) by which caffeine may impair/enhance balance control (as previously mentioned). On a related note, the possibility that caffeine ingestion interacts with cognitive processes to modulate a person’s balance control is also an area that we suggest requires further investigation.

Finally, past research suggests that balance is impaired following prolonged physical and cognitive exertion [53] and sleep deprivation [54]. Increased body sway has also been demonstrated later in the day compared to morning [55]. Therefore, physical and/or mental fatigue, sleep quality and time of day may have been confounding factors in the included studies. Furthermore, as caffeine’s effects may be of relevance to balance control under these conditions, we suggest that future research should investigate whether caffeine can modulate the effects of physical fatigue, mental fatigue, sleepiness and time of day on the control of upright stance.

### 4.4. Implications for Practice

The findings of this systematic review are of importance for several reasons. Firstly, our main finding that caffeine ingestion increases body sway in older adults has considerable implications for today’s ageing population. As increased sway during upright stance, particularly in the mediolateral direction, has previously been linked to risk of falling [56], our finding suggests caffeine ingestion could contribute to falls in old age. A lack of data concerning lower caffeine doses (i.e., <200 mg) means it is not currently possible to determine whether this negative effect would occur following a single serving of tea or coffee. Nonetheless, in light of the potential to increase fall risk, it would be sensible for older adults to consume caffeine only in moderation. Secondly, our findings suggest that pre-experiment caffeine ingestion may be a confounding variable when assessing balance control, particularly in older adults. Therefore, practitioners and researchers should consider prohibiting, limiting or controlling for pre-experiment caffeine ingestion before measuring a person’s body sway. Thirdly, it is possible that caffeine ingestion could be used to enhance balance control in certain circumstances, although more research is needed to fully understand this potential effect. Nevertheless, our findings suggest that younger people seeking ergogenic benefits of caffeine in terms of athletic performance can do so in the knowledge that—while its efficacy to improve balance is currently unclear—there appears to be little to no chance of a detrimental effect on the control of standing in this population.

## 5. Conclusions

Our findings indicate an age-dependent effect of caffeine ingestion on human balance; generally, caffeine had no influence in younger participants but impaired balance in the older age groups. Although caffeine ingestion improved balance control in only one included study, our findings do raise the possibility that balance may be enhanced during more challenging conditions. While the mechanism(s) which underlie these effects require further investigation, in practical terms the findings suggest caffeine ingestion may contribute to poor balance and falls in older adults.

## Figures and Tables

**Table 1:** PICOS method used to formulate eligibility criteria.

PICOS Component	Criterion
Population	Human participants with no exclusions based on age, gender or health status
Intervention	Caffeine ingestion in any form
Comparison	Placebo control
Outcome	Measures of balance control during upright standing
Study design	Crossover or parallel, randomised or non-randomised, placebo-controlled trials

**Table 2:** Search strategy development process.

Search Number	Search Terms	Database			
		Embase	PubMed/MEDLINE	SPORTDiscus	Web of Science
(15)	(“Caffeine” OR “Caffeinated” OR “Energy drinks” OR “Energy drink” OR “Coffee”) AND (“Balance” OR “Postural stability” OR “Postural sway” OR “Static balance” OR “Posture” OR “Postural control” OR “Standing” OR “Upright stance”)	1044	605	138	926
(14)	“Balance” OR “Postural stability” OR “Postural sway” OR “Static balance” OR “Posture” OR “Postural control” OR “Standing” OR “Upright stance”	389,636	409,167	60,518	732,945
(13)	“Upright stance”	537	540	196	837
(12)	“Standing”	91,477	79,613	14,351	157,643
(11)	“Postural control”	6809	6471	3042	10,433
(10)	“Posture”	39,863	85,822	22,218	52,414
(9)	“Static balance”	1176	1145	705	1572
(8)	“Postural sway”	2485	2502	990	3348
(7)	“Postural stability”	3640	3250	1454	4986
(6)	“Balance”	275,715	267,320	30,810	538,394
(5)	“Caffeine” OR “Caffeinated” OR “Energy drinks” OR “Energy drink” OR “Coffee”	63,704	49,123	6147	72,355
(4)	“Coffee”	17,314	16,606	2154	36,579
(3)	“Energy drink” OR “Energy drinks”	1969	1619	1237	2281
(2)	“Caffeinated”	1439	1226	292	1265
(1)	“Caffeine”	49,311	34,878	3461	39,350

**Table 3:** QualSyst assessment of study quality and risk of bias [24].

Study	Question Described	Appropriate Study Design	Appropriate Subject Selection	Characteristics Described	Random Allocation	Researchers Blinded	Subjects Blinded	Outcome Measures Well Defined and Robust to Bias	Sample Size Appropriate	Analytic Methods Well Described	Estimate of Variance Reported	Controlled for Confounding	Results Reported in Detail	Conclusion Supported by Results	Rating
Franks et al. (1975) [25]	1	2	1	2	1	0	2	2	2	1	1	1	1	2	Moderate
Nuotto et al. (1982) [26]	1	2	1	2	1	2	2	2	1	1	1	1	1	2	Moderate
Swift and Tiplady (1988) [27]	2	2	1	2	1	2	2	2	0	1	1	1	1	2	Moderate
Liguori and Robinson (2001) [28]	2	2	2	2	1	2	2	2	1	1	2	2	1	2	Strong
Norager et al. (2005) [29]	2	2	2	2	2	2	2	2	2	2	2	2	2	2	Strong
Momsen et al. (2010) [30]	2	2	2	2	2	2	2	2	2	2	2	2	2	2	Strong
Jensen et al. (2011) [31]	2	2	2	2	2	2	2	2	2	2	2	2	2	2	Strong
Ben Waer et al. (2020) [32]	2	2	1	2	0	2	2	2	2	2	1	1	2	2	Strong
Tallis et al. (2020) [33]	2	2	1	2	1	2	2	2	1	2	1	1	1	2	Strong

Individual item scores: 2 indicates yes, 1 indicates partial, 0 indicates no. Overall rating: ≥55% moderate, ≥75% strong.

**Table 4:** Summary of the included studies with younger participants.

Authors (Date of Publication)	Country	Participant Characteristics	Study Design	Caffeine Abstinence Protocol	Caffeine Condition(s)	Placebo Condition	Balance Measurement Timing (Post-Ingestion)	Balance Measurement Type	Balance Outcome (i.e., Effect of Caffeine)
Franks et al. (1975) [25]	Australia	68 healthy participants 31 M, 37 F 20–28 years ^1^	Randomised parallel	Caffeinated beverages before arrival (duration not reported)	Sugar-free orange squash ^2^ followed by 300 mg/70 kg (i.e., 4.3 mg∙kg^−1^) in de-caffeinated coffee	Sugar-free orange squash ^2^ followed by decaffeinated coffee alone	20, 80 and 140 min	Body sway EO, EC	↑Body sway (EO 20 min, 27% ^3^) EO 80 and 140 min, ns EC 20, 80 and 140 min, ns
Nuotto et al. (1982) [26]	Finland	10 healthy participants 10 M, 0 F 21.1 ± 1.7 years	Randomised double-blind crossover	Caffeinated beverages for 24 h	Pellegrino beverage ^2^ followed by 500 mg in decaffeinated coffee (2 x 250 mg doses separated by 45 min)	Pellegrino beverage ^2^ followed by decaffeinated coffee alone (2 doses separated by 45 min)	30, 60 and 120 min after first dose	Body sway EO, EC	EO 30, 60 and 120 min, ns EC 30, 60 and 120 min, ns
Swift and Tiplady (1988) [27]	UK	6 healthy participants 2 M, 4 F 18–37 years ^1^	Randomised double-blind crossover	Not reported	200 mg capsule	Matching capsule	60, 120 and 180 min	AP body sway EO	EO 60, 120 and 180 min, ns
Liguori and Robinson (2001) [28]	USA	15 healthy participants 6 M, 9 F 21–45 years ^1^ (mean 32 years)	Randomised double-blind crossover	24 h	(a) 200 mg capsule (b) 400 mg capsule followed by orange juice ^2^	Methylcellulose capsule followed by orange juice ^2^	45 min	AP body sway Composite score based on six conditions of the EquiTest	(a) 200 mg caffeine Composite score, ns (b) 400 mg caffeine Composite score, ns
Ben Waer et al. (2020) [32]	Tunisia	25 healthy participants 0 M, 25 F 53 ± 4 years	Double-blind crossover	12 h	(a) 100 mg capsule (b) 400 mg capsule	Empty capsule	30 min	COP_VelMean_ COP_MLpath_ COP_APpath_ EO, EC, EOF, ECF	(a) 100 mg caffeine: ↓COP_VelMean_, ↓COP_MLpath_ and ↓COP_APpath_ (ECF all −16% ^3^) EO, EC and EOF, ns (b) 400 mg caffeine: EO, EC, EOF and ECF, ns

^1^ range reported where mean ± SD not available; ^2^ non-alcoholic placebo (comparison to alcohol condition not reported here); ^3^ calculated using data obtained via WebPlotDigitizer software; AP: anteroposterior; EO: eyes open on firm surface; EC: eyes closed on firm surface; EOF: eyes open on foam surface; ECF: eyes closed on foam surface; COP_VelMean_: mean centre of pressure velocity; COP_MLpath_: mediolateral centre of pressure path length; COP_APpath_: anteroposterior centre of pressure path length; ns: not significant

**Table 5:** Summary of the included studies with older participants.

Authors (Date of Publication)	Country	Participant Characteristics	Study Design	Caffeine Abstinence Protocol	Caffeine Condition(s)	Placebo Condition	Balance Measurement Timing (Post-Ingestion)	Balance Measurement Type	Balance outcome (i.e., Effect of Caffeine)
Swift and Tiplady (1988) [27]	UK	6 healthy participants 3 M, 3 F 65–75 years ^1^	Randomised double-blind crossover	Not reported	200 mg capsule	Matching capsule	60, 120 and 180 min	AP body sway EO	↑AP body sway (EO 180 min) EO 60 and 120 min, ns
Norager et al. (2005) [29]	Denmark	30 healthy participants 15 M, 15 F 74.7 ± 5.5 years	Randomised double-blind crossover	48 h	6 mg∙kg^−1^ capsule	Glucose monohydrate capsule	60 min	COP_VelMom_ EO, EC, EOST	↑COP_VelMom_ (EO 25%, EC 43%) EOST, ns
Momsen et al. (2010) [30]	Denmark	88 patients with intermittent claudication 50 M, 38 F 67.5 ± 6.9 years	Randomised double-blind crossover	48 h	6 mg∙kg^−1^ capsule	Glucose monohydrate capsule	75 min	COP_VelMom_ EO, EC	↑COP_VelMom_ (EO 22%, EC 22%)
Jensen et al. (2011) [31]	Denmark	30 healthy participants 15 M, 15 F 74.1 (70.2–84.9) years ^2^	Randomised double-blind crossover	8 h	6 mg∙kg^−1^ capsule	Glucose monohydrate capsule	60 min	COP_VelMom_ EO, EC, EOST	↑COP_VelMom_ (EO 19%, EC 42%) EOST, ns
Tallis et al. (2020) [33]	UK	12 healthy participants 4 M, 8 F 72 ± 4 years	Randomised double-blind crossover	12 h	3 mg∙kg^−1^ capsule	Maltodextrin capsule	45 min	COP_VelMean_ COP_Path_ COP_MLmax_ COP_APmax_ COP_Ellipse_ EO, EC, EOF, ECF, EO3s, EO7s, EOF3s, EOF7s	↑COP_VelMean_ (EO 21%, EC 25%, EOF 8%, ECF 6%, EO3s 3%, EO7s 8%, EOF3s −6%, EOF7s 9%) ↑COP_Path_ (EO 22%, EC 27%, EOF 8%, ECF 6%, EO3s 2%, EO7s 8%, EOF3s −6%, EOF7s 6%) ↑COP_MLmax_ (EO 89%, EC 114%, EOF 34%, ECF 23%, EO3s 9%, EO7s 93%, EOF3s 6%, EOF7s 2%) COP_APmax_, ns COP_Ellipse_, ns

^1^ range or ^2^ median (5^th^–95^th^ centile) reported where mean ± SD not available; AP: anteroposterior; EO: eyes open on firm surface; EC: eyes closed on firm surface; EOST: eyes open on firm surface in semi-tandem stance; EOF: eyes open on foam surface; ECF: eyes closed on foam surface; EO3s: eyes open on firm surface with concurrent serial threes subtraction task; EO7s: eyes open on firm surface with concurrent serial sevens subtraction task; EOF3s: eyes open on foam surface with concurrent serial threes subtraction task; EOF7s: eyes open on foam surface with concurrent serial sevens subtraction task; COP_VelMom_: mean area covered by centre of pressure per second; COP_VelMean_: mean centre of pressure velocity; COP_Path_: centre of pressure path length; COP_MLmax_: maximal mediolateral centre of pressure displacement; COP_APmax_: maximal anteroposterior centre of pressure displacement; COP_Ellipse_: centre of pressure 95% elliptical area; ns: not significant.

## Data Availability

No new data were created or analysed in this study. Data sharing is not applicable to this article.

## Registration and Protocol

This systematic review protocol was registered in the international prospective register of systematic reviews (PROSPERO) under the registration number CRD42021226939.
